# Supplementary material for: A synaptic corollary discharge signal suppresses midbrain visual processing during saccade-like locomotion
Source: Nat Commun. 2023 Nov 23;14:7592. doi: 10.1038/s41467-023-43255-6 (PMC10667368; doi:10.1038/s41467-023-43255-6)
Supplement: Supplementary file 1 — Supplementary Information [file 41467_2023_43255_MOESM1_ESM.pdf]

Supplementary Information

Supplementary Figures 1-5 and Legends

Supplementary References

A Synaptic Corollary Discharge Signal Suppresses Midbrain Visual Processing During Saccade-Like Locomotion

Mir Ahsan Ali, Katharina Lischka, Stephanie J. Preuss, Chintan A. Trivedi, Johann H. Bollmann

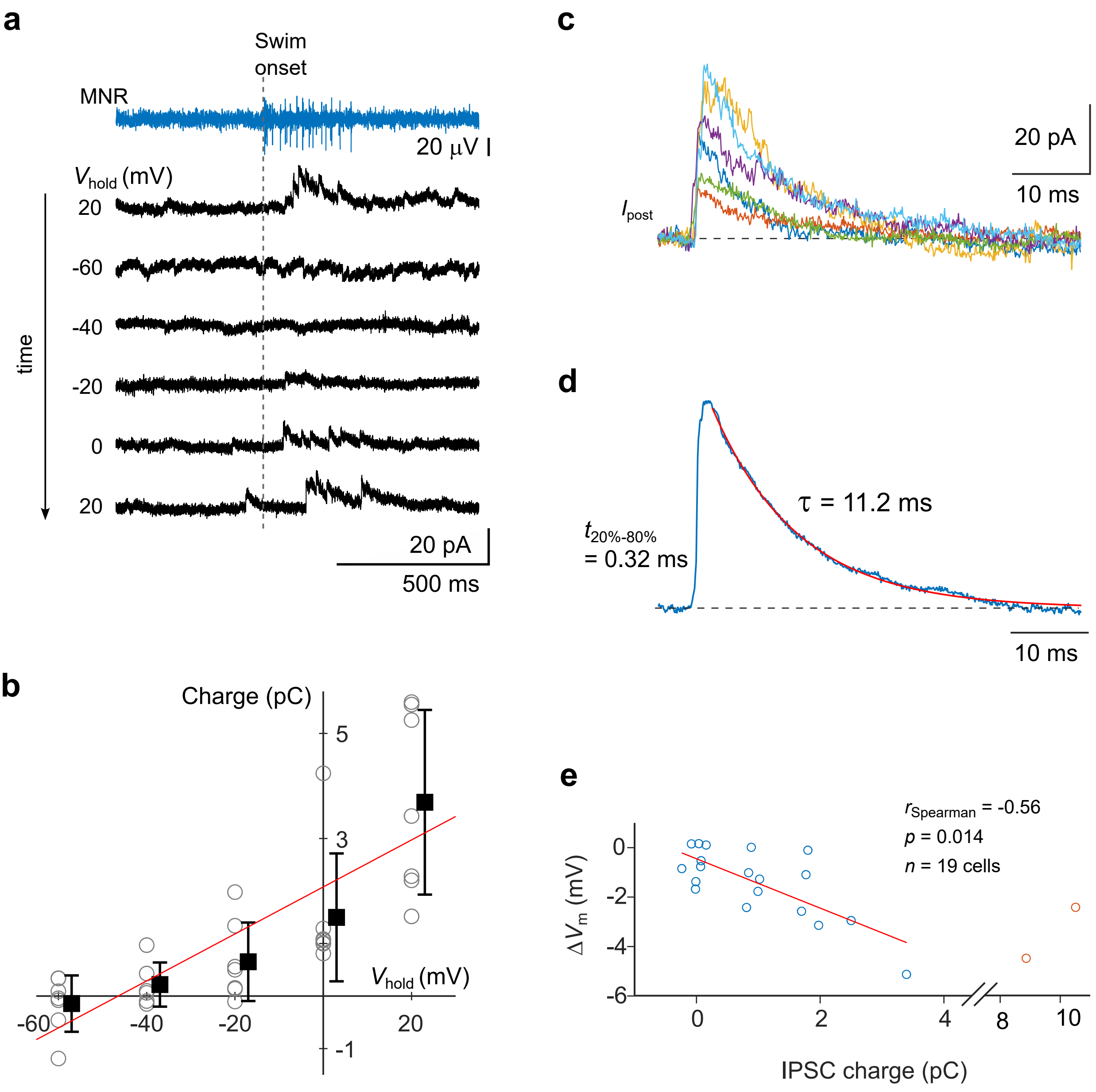

Supplementary Figure 1

Supplementary Figure 1 | Properties of swim-related postsynaptic currents are consistent with GABAergic inhibitory synaptic conductance.

a Examples of membrane currents (black traces) during spontaneous swim activity at different holding potentials (V_{hold}), measured in one cell. Holding potentials were -60 mV, -40 mV, -20 mV, 0 mV and 20 mV. Recovery of the swim-related outward current at positive potentials towards the end of the experiment showed that the reduction in current amplitude was not caused by synaptic run-down. Blue trace: motor nerve recording (MNR) of a spontaneous swim bout. Swim onset indicated by dashed line.

b Relationship between inhibitory charge transfer and membrane potential at five different holding potentials (n = 7 cells; mean ± SD; -60 mV: -0.14 pC ± 0.54 pC; -40 mV: 0.22 pC ± 0.42 pC; -20 mV: 0.65 pC ± 0.75 pC; 0 mV: 1.5 pC ± 1.2 pC; 20 mV: 3.7 pC ± 1.8 pC). Squares and error bars: mean ± SD; red line: linear fit. The negative reversal potential of the charge-voltage-relationship is consistent with an inhibitory GABA-mediated chloride conductance.

c Six example traces of unitary swim-related postsynaptic currents used for fitting the average trace shown in (**d**).

d Average trace of unitary postsynaptic currents (from n = 48 individual traces), measured at V_{hold} = 10 mV. Traces were aligned to the point of maximum slope during the rising phase, peak-scaled, and then averaged. 20-80% rise time of the average trace was 0.32 ms, the time constant of a single-exponential fit to the decay phase (red curve) was 11.2 ms, consistent with the kinetics of GABAergic synaptic conductances[1,2,3].

e Scatter plot of swim-related transient hyperpolarization measured in current clamp (ΔV_m) and IPSC charge transfer measured in voltage clamp. Same data as in Fig. **2e**, but with a linear fit that excludes the two rightmost data points. The swim-related hyperpolarization measured in current clamp and the swim-related IPSC charge are negatively correlated (n = 19 cells, p = 0.014, Spearman rank correlation).

Source data are provided as a Source Data file.

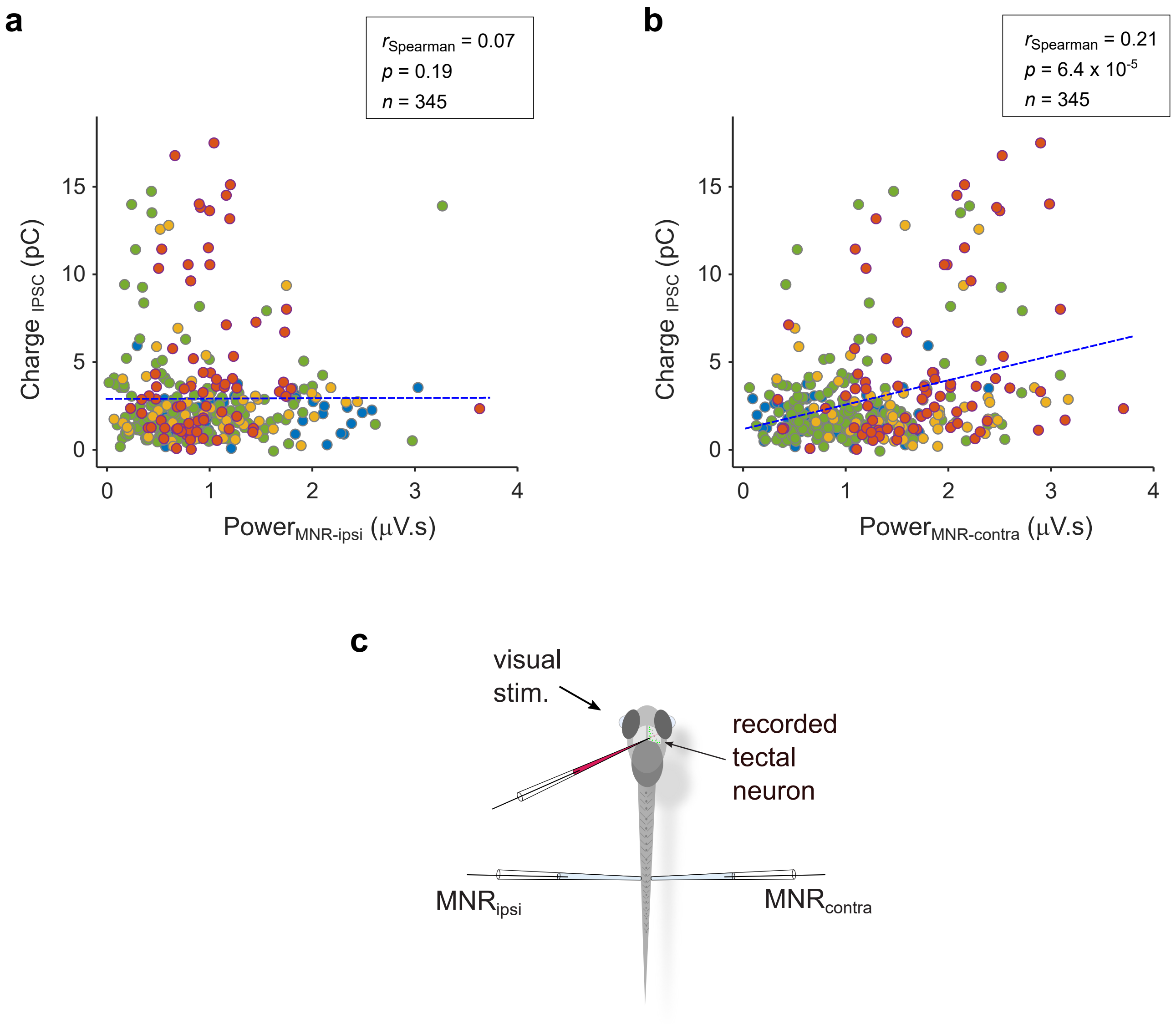

Supplementary Figure 2

Supplementary Figure 2 | Swim-related inhibitory charge in tectal cells is correlated with tail swim power measured on the same side.

a Scatter plot of IPSC charge transfer associated with spontaneous swims (green) and swims evoked by small rectangles (blue), large rectangles (yellow) and expanding discs (red). x-axis represents swim power measured on the side of the tail opposite to the recorded neuron (that is, ipsilateral to the visual stimulus, MNR_{ipsi}). Charge transfer was uncorrelated to swim power (n = 345 swims, p = 0.19, Spearman rank correlation). Same data as in Fig. **3h**.Swim power was quantified as described in Fig. **3c**.

b Scatter plot of IPSC charge transfer and swim power on the same side of the tail as the recorded neuron, that is, contralateral to the visual stimulus (MNR_{contra}). IPSC charge is correlated to swim power on this side (n = 345 swims, $p = 6.4 \times 10^{-5}$, Spearman rank correlation).

c Schematic of the recording configuration, for clarity.

Source data are provided as a Source Data file.

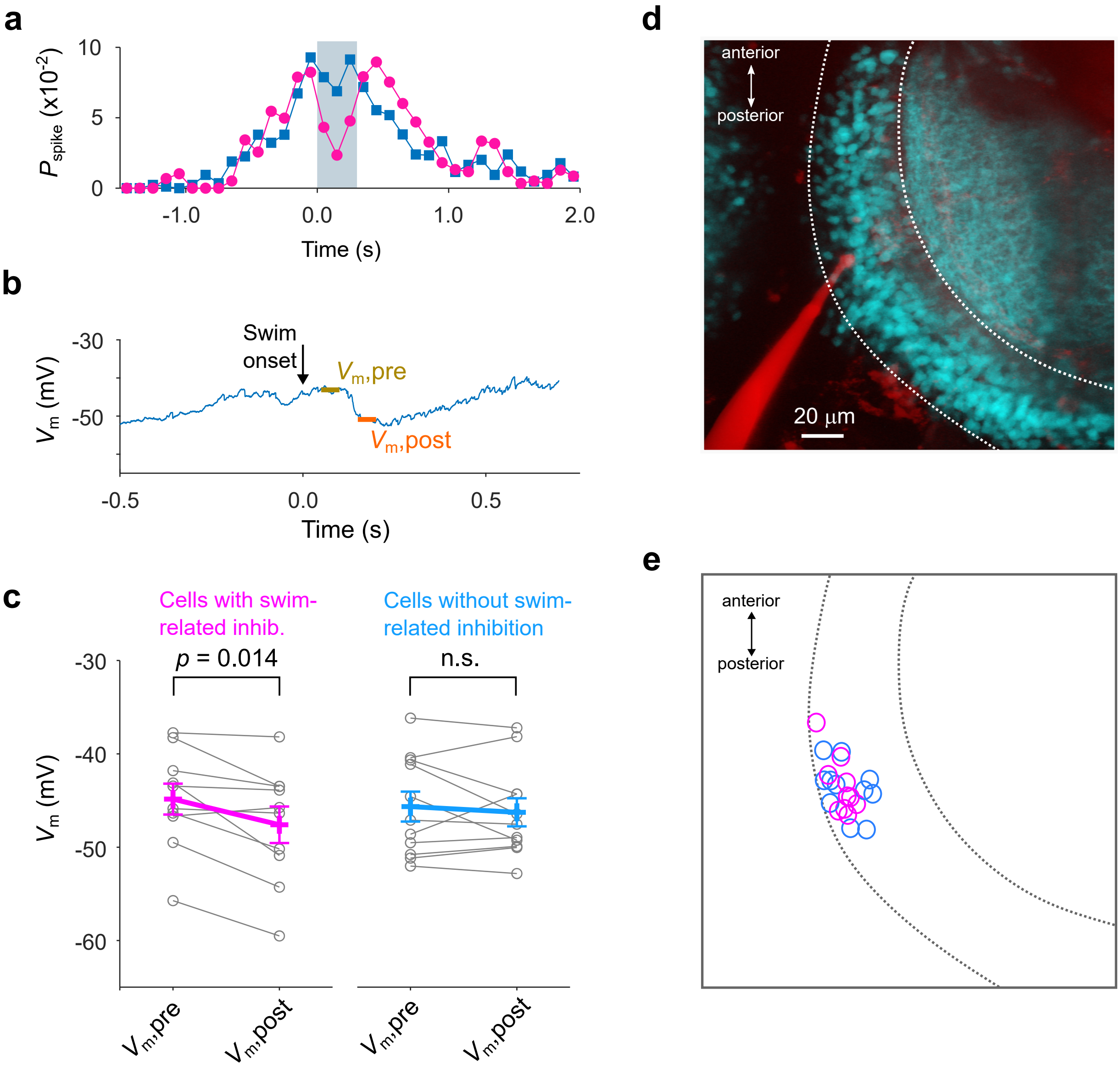

Supplementary Figure 3

Supplementary Figure 3 | Effects of swim-related inhibition on spike probability and membrane voltage during visual stimulation

a Time course of the population spike probability (P_{spike}) aligned to swim onset during visual stimulation with expanding discs, for the cell population with detectable inhibitory synaptic charge transfer (magenta) and that with negligible charge transfer (blue). Note the strong transient reduction in spike probability in a ~300 ms window (grey bar) in the magenta trace, following the onset of swimming. Time course of P_{spike} of the two populations was calculated as the number of spikes counted across each population per 100 ms-bin, divided by the total number of spikes summed across a time window [-1.5 s, 2.0 s] around the swim event.
Same data as in Fig**. 4e-g**.

b Example of membrane voltage during visual stimulation with expanding disc. Visually evoked spikes were removed by interpolation between the onset and offset of a spike. The change in membrane potential following the onset of a visually driven swim bout was quantified by averaging the membrane voltage in the indicated windows ($V_{m,pre}$: 50-100 ms following swim onset; $V_{m,post}$: 150-200 ms following swim onset).

c Comparison of membrane voltage change ($\Delta V_m = V_{m,post} - V_{m,pre}$) in each cell following swim onset for the cell population with detectable inhibitory synaptic charge transfer (magenta) and that with negligible charge transfer (blue). Individual cell data (grey circles) and averages ± SEM (magenta and blue crosses and error bars, respectively). Membrane voltage in cells with swim-related inhibition is significantly hyperpolarized: ΔV_m = - 2.8 ± 0.9 mV (mean ± SEM, n = 10 cells, p = 0.014, two-sided Wilcoxon signed-rank test for paired samples), but not in cells with negligible charge transfer (<0.8 pC): ΔV_m = -0.6 ± 0.9 mV, n = 11 cells, p = 0.7, two-sided Wilcoxon signed-rank test for paired samples).

d Fluorescence image of a recorded neuron (red) in *Tg(pou4f1-hsp70l:GFP)* transgenic background (cyan), illustrating the cell's position in the periventricular cell body layer (PVL, outlined by white dotted curves). Micrograph representative of n = 21 independent experiments.

e Positions of recorded neurons. Neurons with detectable inhibitory synaptic charge transfer: magenta (n = 10). Neurons with negligible charge transfer: blue (n = 11). Neurons were typically recorded in the central sector of the PVL.

Source data are provided as a Source Data file.

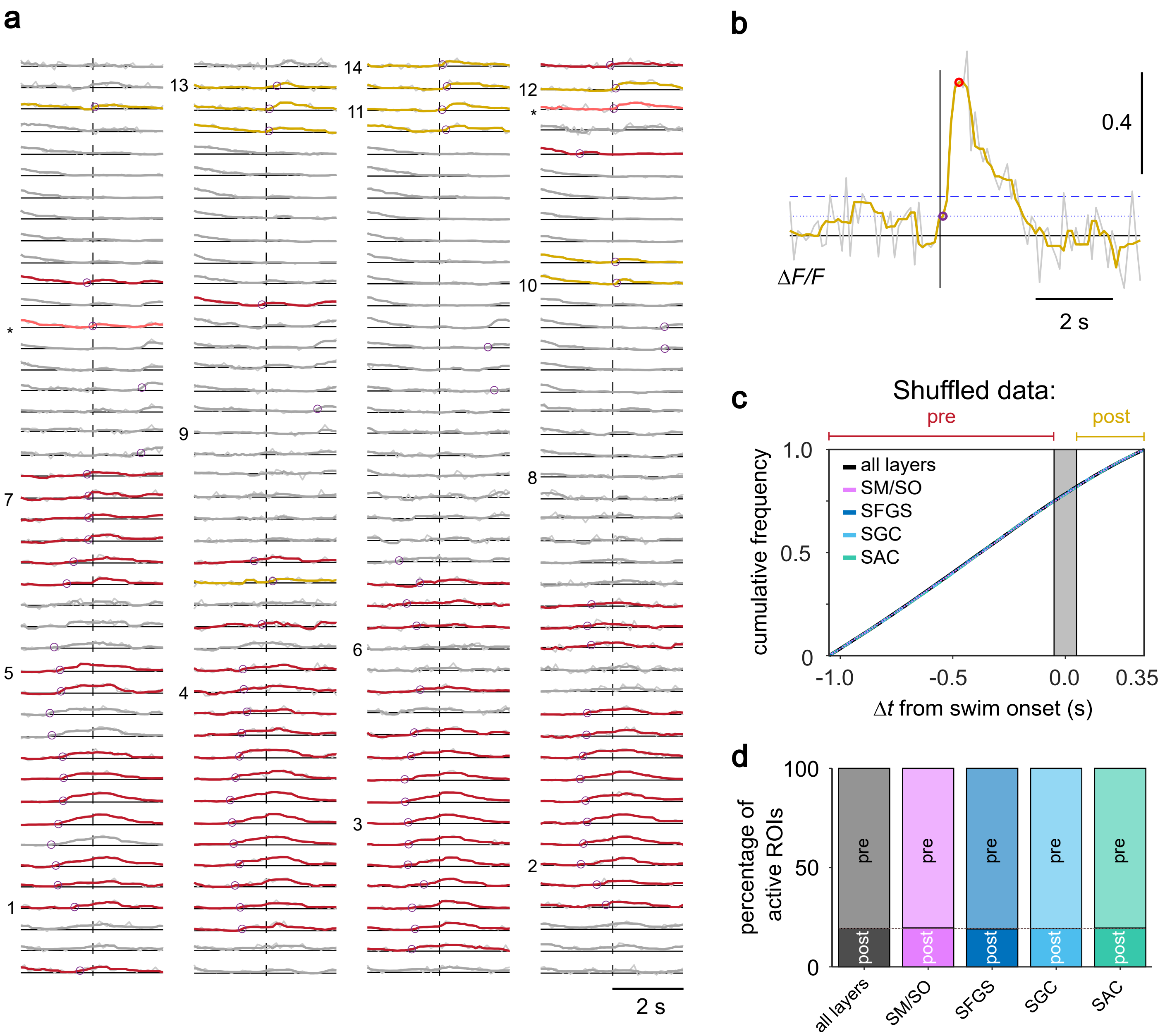

Supplementary Figure 4

Supplementary Figure 4 | Spatially resolved measurement of Ca2+ transients in the tectal neuropil during spontaneous swim bouts

a Array of 172 $\Delta F/F$ traces across the tectal neuropil during a spontaneous swim bout measured in equally spaced square-shaped ROIs (numbers indicate traces shown in Fig. **6c**; same data as in Fig. **6c**). Swim onset indicated by vertical line. Colored traces indicate ROIs with a supra-threshold peak $\Delta F/F$ value in the interval [-1.05 s; 0.35 s] around swim onset (see Methods). Red traces indicate ‘pre-swim’ $\Delta F/F$ transients in the interval [-1.05 s; -0.05 s], yellow traces indicate ‘post-swim’ $\Delta F/F$ transients in the interval [0.05 s; 0.35 s]. The onset of some traces fell in the interval [-0.05 s; 0.05 s] for which assignment to the ‘pre’ or ‘post’ $\Delta F/F$ classes was not done (asterisks). All other $\Delta F/F$ traces with peaks outside the stated time interval or without a significant peak shown in grey. Circles mark the onset of $\Delta F/F$ transients, where the signal crosses a 3-standard deviation threshold. All $\Delta F/F$ traces were individually peak-scaled for clarity.

b Analysis of neuropil $\Delta F/F$ transients. The raw $\Delta F/F$ trace (grey) was filtered with a moving median window of length 5 (yellow trace). The baseline was measured as the mean of the 60% lowest points of the filtered trace (black horizontal line). The standard deviation (SD) was calculated from the 33% lowest points of the filtered trace. All peaks of the filtered trace that exceeded a 6-SD threshold (horizontal dashed line) and had a width ≥4 sample points were taken as Ca2+ transients. The peak closest to the swim onset (vertical black line) was selected (red circle) and the time of the $\Delta F/F$ trace’s crossing the 3-SD-level (horizontal dotted line) was taken as the Ca2+ transient onset (dark circle). If the transient onset occurred in an interval [-1.05 s; 0.35 s] around the swim, the transient was considered to reflect pre-motor activity or a potential corollary discharge signal in the tectal neuropil (‘active ROI’, compare Fig. **6d**).

c Cumulative distribution of active ROIs in shuffled data (using traces from hundred separate rounds of shuffling the original data). Active ROIs were pooled over all layers (black trace) and pooled separately for the different neuropil regions (colored traces, analogous to Fig. **6f**). The cumulative distributions for individual regions were not different from that representing ROIs from all layers (SM/SO: p = 1.0; SFGS: p = 0.56; SGC: p = 1.0; SAC: 0.056, Kolmogorov-Smirnov tests with Bonferroni adjustment of p-values for multiple comparisons).

d Fractions of active ROIs from the [-1.05 s; -0.05 s] interval classified as ‘pre-swim’ and those in the [0.05 s; 0.35 s] interval classified as ‘post-swim’, pooled over all layers (left bar) and pooled separately for the different neuropil regions (colored bars). The fractions of post-swim active ROIs determined for the individual layers were not different from that counted across all layers (SM/SO: p = 0.236; SFGS: p = 0.192; SGC: p = 1.0; SAC: p = 0.052, respectively; two-sided binomial tests with Bonferroni adjustment of p-values for multiple comparisons).

Source data are provided as a Source Data file.

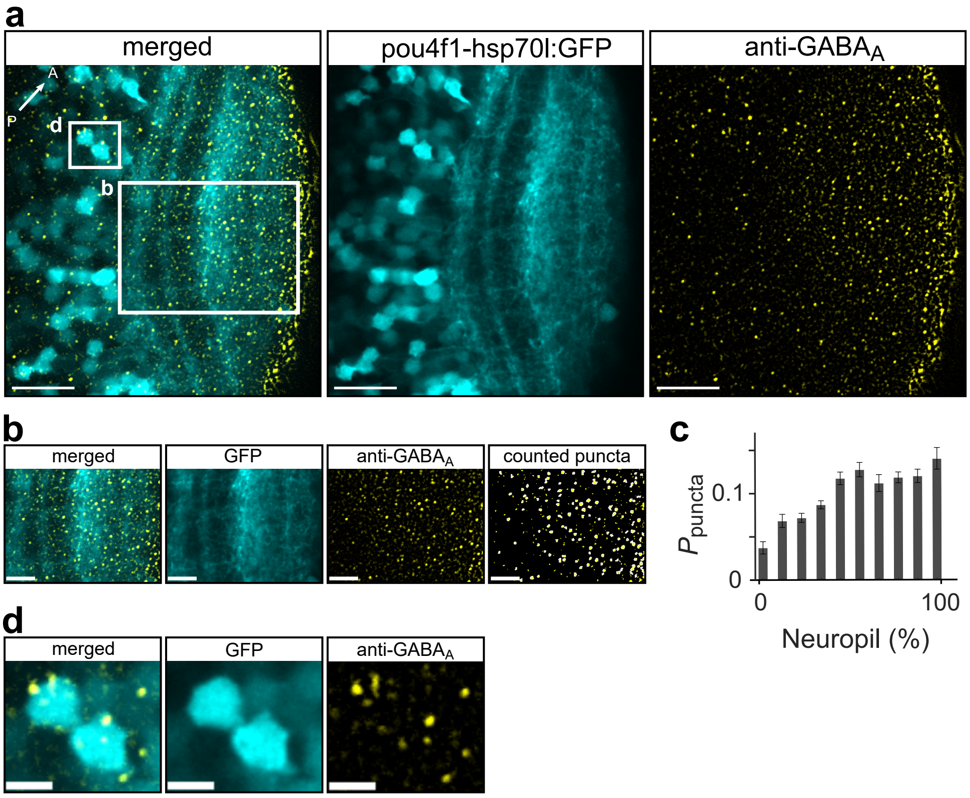

Supplementary Figure 5

Supplementary Figure 5 | Distribution of $GABA_A$ receptor subunits

a Single optical section of GFP fluorescence and immunostaining of a tectal hemisphere in a 5 dpf larva (dorsal view). *Middle*: GFP expression in *Tg(pou4f1-hsp70l:GFP)*. *Right*: anti-$GABA_A$ receptor immunostaining. *Left*: merged image of both channels. Scale bar 20 µm. Micrographs representative of *n* = 7 independent experiments.

b Neuropil region indicated by large box in (**a**) showing the merged (left), the GFP stratification pattern in the neuropil (middle left), and localization of $GABA_A$-positive puncta (middle right) in the neuropil. Puncta exceeding thresholds for size and circularity (marked with a white outline in rightmost panel) were counted as $GABA_A$ receptor clusters representing putative inhibitory synapses and included in the histogram in (**c**). Scale bar 10 µm.

c Histogram of relative abundance of $GABA_A$-positive puncta in the tectal neuropil as a function of neuropil depth (*n* = 11 samples from 7 fish; mean ± SEM; 0-10%: 0.0373 ± 0.0072; 10-20%: 0.0684 ± 0.0076; 20-30%: 0.0719 ± 0.0052; 30-40%: 0.0869 ± 0.0046; 40-50%: 0.1174 ± 0.0073; 50-60%: 0.1275 ± 0.0082; 60-70%: 0.1118 ± 0.0098; 70-80%: 0.1184 ± 0.0062; 80-90%: 0.1201 ± 0.0078; 90-100%: 0.1403 ± 0.0125). X-axis: 0% corresponds to PVL/neuropil boundary, 100% to dorsal neuropil boundary, bin width 10%.

d Magnified view of region indicated by box in (**a**) showing the merged (left), the GFP expression (middle), and $GABA_A$–positive puncta (right). Note colocalization of $GABA_A$ receptor-positive puncta and surface of GFP-positive cell bodies. Scale bar 5 µm.

Source data are provided as a Source Data file.

Supplementary References

1 Xiang, Z., Huguenard, J. R. & Prince, D. A. GABAA receptor-mediated currents in interneurons and pyramidal cells of rat visual cortex. *J Physiol* **506 (Pt 3)**, 715-730 (1998).

2 Gupta, A., Wang, Y. & Markram, H. Organizing principles for a diversity of GABAergic interneurons and synapses in the neocortex. *Science* **287**, 273-278 (2000).

3 Bartos, M. *et al.* Fast synaptic inhibition promotes synchronized gamma oscillations in hippocampal interneuron networks. *Proc Natl Acad Sci U S A* **99**, 13222-13227 (2002).
