# Supplementary material for: A synaptic corollary discharge signal suppresses midbrain visual processing during saccade-like locomotion
Source: Nat Commun. 2023 Nov 23;14:7592. doi: 10.1038/s41467-023-43255-6 (PMC10667368; doi:10.1038/s41467-023-43255-6)
Supplement: Supplementary file 3 — Reporting Summary [file 41467_2023_43255_MOESM3_ESM.pdf]

nature portfolio

Corresponding author(s): Johann H Bollmann

Last updated by author(s): Oct 23, 2023

Reporting Summary

Nature Portfolio wishes to improve the reproducibility of the work that we publish. This form provides structure for consistency and transparency in reporting. For further information on Nature Portfolio policies, see our Editorial Policies and the Editorial Policy Checklist.

Statistics

For all statistical analyses, confirm that the following items are present in the figure legend, table legend, main text, or Methods section.

n/a	Confirmed	
☐	☒	The exact sample size (*n*) for each experimental group/condition, given as a discrete number and unit of measurement
☐	☒	A statement on whether measurements were taken from distinct samples or whether the same sample was measured repeatedly
☐	☒	The statistical test(s) used AND whether they are one- or two-sided<br>*Only common tests should be described solely by name; describe more complex techniques in the Methods section.*
☐	☒	A description of all covariates tested
☐	☒	A description of any assumptions or corrections, such as tests of normality and adjustment for multiple comparisons
☐	☒	A full description of the statistical parameters including central tendency (e.g. means) or other basic estimates (e.g. regression coefficient) AND variation (e.g. standard deviation) or associated estimates of uncertainty (e.g. confidence intervals)
☐	☒	For null hypothesis testing, the test statistic (e.g. *F*, *t*, *r*) with confidence intervals, effect sizes, degrees of freedom and *P* value noted<br>*Give P values as exact values whenever suitable.*
☒	☐	For Bayesian analysis, information on the choice of priors and Markov chain Monte Carlo settings
☒	☐	For hierarchical and complex designs, identification of the appropriate level for tests and full reporting of outcomes
☒	☐	Estimates of effect sizes (e.g. Cohen's *d*, Pearson's *r*), indicating how they were calculated

Our web collection on statistics for biologists contains articles on many of the points above.

Software and code

Policy information about availability of computer code

Data collection	Electrophysiological data were acquired using scripts written in LabVIEW (2013 - 2021, National Instruments).<br>Calcium imaging data and images of immunolabeled samples were acquired using Olympus Fluoview Software Version 4.2a.<br>Visual stimuli were programmed in Python 2.7, using the open source library 'VisionEgg' (Straw AD, Front. Neuroinform 2008).<br>Calcium imaging and motor nerve recordings were synchronized using a trigger programmed in Python 2.7.
Data analysis	Electrophysiological data were analyzed using scripts written in LabVIEW (2013 - 2021, National Instruments, USA) and Matlab (2021a, The MathWorks, USA). Calcium imaging data were analyzed using code written in Matlab (2021a, The MathWorks).<br>Analysis of neuron morphology and antibody labeling was performed using ImageJ 1.53, in the FIJI distribution package.<br>Code used for analysis of Ca2+ imaging data is available at https://github.com/bollmannlab/Corollary_Discharge/.<br>Other scripts used for data acquisition and analysis are available upon request to the corresponding author.

For manuscripts utilizing custom algorithms or software that are central to the research but not yet described in published literature, software must be made available to editors and reviewers. We strongly encourage code deposition in a community repository (e.g. GitHub). See the Nature Portfolio guidelines for submitting code & software for further information.

Data

Policy information about availability of data

All manuscripts must include a data availability statement. This statement should provide the following information, where applicable:

- Accession codes, unique identifiers, or web links for publicly available datasets
- A description of any restrictions on data availability
- For clinical datasets or third party data, please ensure that the statement adheres to our policy

The source data for all figures and supplementary figures in this work are provided with this paper. Raw data are available upon request to the corresponding author. Source data are provided with this paper.

Research involving human participants, their data, or biological material

Policy information about studies with human participants or human data. See also policy information about sex, gender (identity/presentation), and sexual orientation and race, ethnicity and racism.

Reporting on sex and gender	The research in the current study did not involve human subjects.
Reporting on race, ethnicity, or other socially relevant groupings	See above.
Population characteristics	See above.
Recruitment	See above.
Ethics oversight	See above.

Note that full information on the approval of the study protocol must also be provided in the manuscript.

Field-specific reporting

Please select the one below that is the best fit for your research. If you are not sure, read the appropriate sections before making your selection.

☒ Life sciences ☐ Behavioural & social sciences ☐ Ecological, evolutionary & environmental sciences

For a reference copy of the document with all sections, see nature.com/documents/nr-reporting-summary-flat.pdf

Life sciences study design

All studies must disclose on these points even when the disclosure is negative.

Sample size	Sample size estimates were determined based on previous studies from our own work (Gabriel et al 2012, Preuss et al, 2014) and that of others and are comparable to similar, previous publications in the field.
Data exclusions	For whole-cell patch clamp recordings of inhibitory currents, data from cells were only included if the larva exhibited spontaneous fictive swim activity while the cell was held in voltage clamp mode, so that we could determine whether it received inhibitory synaptic inputs during spontaneous swimming (Fig. 2). Recordings from 56 cells met this criterion. For 21 cells out of this dataset, it was also possible to obtain membrane voltage recordings in current clamp mode while the fish exhibited spontaneous swims (Fig. 1). Also, for 21 out of the 56 cells, it was possible to measure spiking activity in current clamp mode during visual looming-evoked swim activity (Fig. 4).<br>For calcium imaging of active ROIs in the neuropil (Fig. 6) and in torus longitudinalis neurons (Fig. 7), recordings from fish were excluded that exhibited no spontaneous fictive swim activity, or if motion artifacts during imaging precluded the identification of calcium transients in subsequent analysis.<br>Additional experiments were performed on 24 neurons (Fig. 5) in which visually driven excitation was measured. In 7 of these 24 neurons, we confirmed that these cells also received swim-related inhibition, while this was not addressed in the remaining 17 cells.
Replication	Multiple independent samples were collected for each experiment. The findings were reproducible across samples taken from individual larvae from different clutches on different days. No more than one tectal neuron was recorded from individual larvae. Specifically, experiments were replicated in the following way: Measurement of motor-related inhibitory currents during spontaneous and visually evoked swimming, n = 56 cells (Fig. 2 and 3). Measurement of spiking activity during looming-evoked swims, n = 21 cells (Fig. 4). Measurement of visually evoked excitatory currents, n = 24 (Fig. 5). Measurement of tectal Ca^{2+} signals during spontaneous swimming, n = 15 larvae (Fig. 6). Measurement of somatic Ca^{2+} signals in torus longitudinalis, n = 5 larvae (Fig. 7). Measurement of dendritic stratification profiles, n = 56 cells (Fig. 8, same cells as in Fig. 2). Measurement of charge-voltage-relationship of motor-related synaptic inputs, n = 7 (Suppl. Fig. 1a,b). Measurement of distribution of GABA-A receptor subunits, n = 7 fish (Suppl. Fig. 5).
Randomization	For all experiments, larvae were selected at random from clutches that exhibited spontaneous swimming activity. For whole-cell patch clamp

Randomization	recordings, tectal cells were selected for GFP-expression/no expression, but otherwise at random from the central area of the tectal periventricular cell body layer. For the measurement of tectal Ca2+ signals during spontaneous swimming, larvae were selected based on the presence of GCaMP5 expression in the nervous system, but otherwise at random.<br>When measuring synaptic currents and tectal Ca2+ signals during spontaneous swimming, randomization is not relevant because of the stochastic nature of spontaneous swim events. When measuring synaptic currents during different visual stimulus types (Fig. 3) or at different holding potentials (Fig. 5 and Suppl. Fig.1), visual stimuli (or holding potentials) were presented in pseudo-random order.
Blinding	Blinding during data collection was not relevant because we performed electrophysiology from randomly selected neurons and Ca2+ imaging in randomly selected transgenic larvae of uniform genotype, as opposed to measuring differences across individual larvae, e.g. from different genotypes. Analysis of electrophysiological data was performed blind to the morphology of each neuron. Electrophysiological and fluorescence signals were analyzed using consistent settings in analysis software and therefore blind across animals.

Reporting for specific materials, systems and methods

We require information from authors about some types of materials, experimental systems and methods used in many studies. Here, indicate whether each material, system or method listed is relevant to your study. If you are not sure if a list item applies to your research, read the appropriate section before selecting a response.

Materials & experimental systems

n/a	Involved in the study
☐	☒ Antibodies
☒	☐ Eukaryotic cell lines
☒	☐ Palaeontology and archaeology
☐	☒ Animals and other organisms
☒	☐ Clinical data
☒	☐ Dual use research of concern
☒	☐ Plants

Methods

n/a	Involved in the study
☒	☐ ChIP-seq
☒	☐ Flow cytometry
☒	☐ MRI-based neuroimaging

Antibodies

Antibodies used	Primary antibodies: mouse anti-GABA (Merck, cat. number MAB341, clone BD17, lot. number 3460348, 1:100); chicken anti-GFP (Invitrogen, cat. number A10262, lot. number 2407370, 1:400)<br>Secondary antibodies: goat anti-mouse 546 (Invitrogen, cat. number A11003, lot. number 2206621, 1:1000); goat anti-chicken 488 (Invitrogen, cat. number A11039, lot. number 2304258, 1:1000)
Validation	Validation was determined by reviewing the manufacturer's literature, other published research, and prior experiments in the lab. For each experiment, at least one slide was designated for a "secondary only" control and examined for potential background staining. Secondary antibodies were validated by the manufacturer.<br>mouse anti-GABA antibody has been validated previously in other teleost species (salmon: Anzelius et al., 1995; trout: Folgueira et al., 2007, Journal of Comparative Neurology)<br>chicken anti-GFP (https://www.thermofisher.com/antibody/product/GFP-Antibody-Polyclonal/A10262)<br>anti-mouse 546 (https://www.thermofisher.com/antibody/product/Goat-anti-Mouse-IgG-H-L-Cross-Adsorbed-Secondary-Antibody-Polyclonal/A-11003)<br>anti-chicken 488 (https://www.thermofisher.com/antibody/product/Goat-anti-Chicken-IgY-H-L-Secondary-Antibody-Polyclonal/A-11039)

Animals and other research organisms

Policy information about studies involving animals; ARRIVE guidelines recommended for reporting animal research, and Sex and Gender in Research

Laboratory animals	Species: Danio rerio, age 5-8 days post-fertilization. Strains used: Tg(pou4f1-hsp70l:GFP), Tg(elavl3:GCaMP5G), Tg(elavl3.1:Gal4-VP16;UAS:GCaMP3), in the nacre background (mitfa-/-; Lister et al, Development 1999).
Wild animals	The study did not involve wild animals.
Reporting on sex	Experiments were performed on zebrafish larvae age 5-8 days post fertilization, that is, before sexual differentiation.
Field-collected samples	The study did not involve wild animals.
Ethics oversight	All procedures were performed following the guidelines of the German animal welfare law and approved by local authorities (Regierungspräsidien Karlsruhe and Freiburg).

Note that full information on the approval of the study protocol must also be provided in the manuscript.
